# Trends towards Lower Susceptibility of *Spodoptera frugiperda* (Lepidoptera: Noctuidae) to Teflubenzuron in Brazil: An Evidence for Field-Evolved Resistance

**DOI:** 10.3390/insects14020129

**Published:** 2023-01-26

**Authors:** Fernando Semmelroth de Assunção e Amaral, Rubens Hideo Kanno, Antonio Rogério Bezerra do Nascimento, Aline Sartori Guidolin, Celso Omoto

**Affiliations:** Department of Entomology and Acarology, Luiz de Queiroz College of Agriculture, University of São Paulo, Piracicaba 13418-900, Brazil

**Keywords:** fall armyworm, field-evolved resistance, chitin synthesis inhibitors, monitoring

## Abstract

**Simple Summary:**

The fall armyworm (FAW), *Spodoptera frugiperda* (J.E Smith), is an important insect pest that can cause severe damage to a wide range of economically important crops. Many insecticides are used to manage the FAW in Brazil, including teflubenzuron. Here, we monitored the susceptibility of the FAW to teflubenzuron in more than 200 field-collected populations from major corn-growing regions of Brazil, from 2004 to 2020. Overall, our findings showed that the FAWs susceptibility to this insecticide reduced over the years, providing evidence of field-evolved resistance. We discuss the possible causes for this loss in the susceptibility to teflubenzuron in *S. frugiperda* and focus on the importance of implementing resistance management strategies.

**Abstract:**

Susceptibility monitoring to insecticides is a key component to implementing insecticide resistance management (IRM) programs. In this research, the susceptibility to teflubenzuron in *Spodoptera frugiperda* (J.E Smith) was monitored in more than 200 field-collected populations from major corn-growing regions of Brazil, from 2004 to 2020. Initially, we defined a diagnostic concentration of 10 µg mL^−1^ of teflubenzuron using a diet-overlay bioassay for monitoring the susceptibility. A variation in the susceptibility to teflubenzuron in *S. frugiperda* was detected among populations from different locations. We also detected a significant reduction in the susceptibility to teflubenzuron throughout time in all the populations of *S. frugiperda* evaluated, with larval survival at diagnostic concentration varying from values of <5% in 2004 to up 80% in 2020. Thus, this research provides evidence of field-evolved resistance of *S. frugiperda* to teflubenzuron and reinforces that IRM practices are urgently needed to be implemented in Brazil.

## 1. Introduction

The fall armyworm (FAW), *Spodoptera frugiperda* (J.E. Smith, 1797) (Lepidoptera: Noctuidae), is a native species from the American continent, but in recent years it has turned into a global relevance pest, due to the rapid invasion of this pest to the Old World countries, causing severe damage mainly to corn (*Zea mays* L.) and cotton (*Gossypium hirsutum* L.) [1,2,3]. The high degree of polyphagia with more than 350 known host species [4] and biological advantages, such as high reproductive and dispersion capacity, favored this pest adaptation in those recently invaded areas [5]. In Brazil, *S. frugiperda* is one of the most important pests in corn, cotton, and soybean (*Glycine max* (L.) Merrill) [4,6,7].

Chemical control and genetically modified plants expressing proteins of *Bacillus thuringiensis* Berliner (Bt) are the main strategies for managing the FAW [8]. Nevertheless, the intensive use of those control methods, associated with the incorrect use of these tools over time, has led to some control failures due to resistance. Approximately 200 resistance cases have been reported to active ingredients in most chemical groups and genetically modified plants expressing proteins from *Bacillus thuringiensis* (Bt) used for *S. frugiperda* control [9]. In Brazil, there is an aggravating situation because of large and intensive cropping systems with diverse and complex insect pests. Therefore, the use of insecticides to control cross-crop insect pests may favor the selection for resistance of *S. frugiperda* to insecticides. Resistance of *S. frugiperda* has already been documented to insecticides and Bt proteins, including pyrethroids, organophosphates, carbamates, chitin synthesis inhibitors, spinosyns, diamides, and Cry1 proteins [10,11,12,13,14,15,16,17,18].

The insecticides in the group of benzoylureas have been widely used to control *S. frugiperda* in Brazil. This chemical group represents 15 to 29% of the insecticide market share to control lepidopteran pests over the last 7 years [19]. Benzoylurea insecticides block correct chitin deposition and cuticle synthesis in insects, acting on the chitin synthase 1 enzymes (CHS1), responsible to catalyze critical stages in insect development [20,21,22,23,24]. These insecticides are well-explored in integrated pest management (IPM) and insect resistance management (IRM) programs due to the low toxicity to mammals and high selectivity to non-target organisms [25]. Teflubenzuron is one of the active ingredients of this group that gained visibility for *S. frugiperda* management, mainly because it provides high activity against lepidopteran and hemipteran pests [23,26]. Because of the high use of benzoylureas in Brazil, cases of resistance have already been reported in *S. frugiperda*, *Chrysodeixis includens* (Lepidoptera: Noctuidae) and *Tuta absoluta* (Lepidoptera: Gelechiidae) [11,27,28,29], evidencing the high risk of resistance evolution to teflubenzuron that could impair the control of FAW.

Understanding the insecticide susceptibility of geographically distinct populations over time is essential to designing insect resistance management (IRM) strategies. The survival pattern of different insect populations can be discussed in association with many variables, such as climate and crop production systems, to show the prone regions to insecticide resistance evolution. In this context, we aimed to describe the spatial and temporal variabilities in the susceptibility to teflubenzuron in *S. frugiperda* field-collected populations from major corn-growing regions of Brazil from 2004 to 2020. This research provides the basis for implementing IRM strategies in Brazil as well as in other countries.

## 2. Material and Methods

### 2.1. Spodoptera frugiperda Populations

The susceptible strain (Sf-ss) used to estimate the diagnostic concentration was obtained from Embrapa Milho e Sorgo (Sete Lagoas, Minas Gerais, Brazil) in 1996, and it has been maintained under laboratory conditions without any selection pressure with insecticides. Populations of *S. frugiperda* used to monitor the susceptibility to teflubenzuron were collected from major corn-growing regions of Brazil on non-Bt cornfields (*Zea mays* L.). Two hundred and sixteen populations were collected from 2004 to 2020 (Table S1). The sample from each population consisted of 600 to 800 larvae. 

Larvae of all populations of *S. frugiperda* were reared in wheat, bean, and yeast-based artificial diet until the pupal stage [30]. Pupae were maintained in PVC cages (24 × 22.5 cm) internally coated with paper, where adults emerge for mating and oviposition. Adults were fed with a 10% honey solution, and, both honey and cage coating paper were replaced every 2 days. Neonates were put in plastic cups (100 mL) containing artificial diet until they reached the third instar, when they were used in bioassays. Insects were kept in controlled conditions of 25 ± 1 °C, 70 ± 10% relative humidity and 14:10 h (L:D) photoperiod during all rearing stages and bioassays.

### 2.2. Bioassays

A diet-overlay bioassay method was used to evaluate the susceptibility of *S. frugiperda* populations to teflubenzuron using 24-well plates (Corning^®^ Costar^®^, Sigma-Aldrich Brasil Ltd.a, São Paulo, Brasil), with 1.25 mL of artificial diet per well (1.9 cm^2^ area) [30]. In each well, 30 µL of teflubenzuron solution was pipetted. Teflubenzuron solutions were obtained by the dilution of the commercial product (Nomolt^®^, 150 g teflubenzuron L^−1^, BASF SA, São Paulo, Brasil) in distilled water with 0.1% (*v*/*v*) Triton^TM^ X-100 (Sigma-Aldrich Brasil Ltd.a.) as surfactant. After drying, a single third-instar larva was placed in each well. Plates were sealed and maintained in a chamber at 25 ± 2 °C, 70 ± 10% RH, and a 14:10 h (L:D) photoperiod. Mortality was assessed at 5 days after insecticide exposure. Larvae that did not show any coordinated movement after being prodded with a brush or failed molting were considered dead.

### 2.3. Concentration-response Curve of S. frugiperda Susceptible Strain to Estimate the Diagnostic Concentration

Seven logarithmically spaced concentrations of teflubenzuron, ranging from 0.1 to 5.6 µg mL^−1^, were used to obtain mortality from 5 to 95% and for the concentration-response curve of Sf-ss strain. The bioassays were conducted in a completely randomized design with four replicates (each replicate was composed of 24 larvae), totaling 96 larvae tested per concentration. 

Data were corrected for controlling mortality using Abbott’s formula [31]. The LC_50_ and LC_99_ values (lethal concentration that kills 50% and 99% of the individuals, respectively), and the respective 95% confidence intervals were estimated using a generalized linear model (GLM) with binomial distribution as formula family and logit as the link function in the R software [32]. The diagnostic concentration used for monitoring the susceptibility of *S. frugiperda* populations to teflubenzuron was based on a concentration close to the upper limit of LC_99,_ estimated from the concentration-response curve of the Sf-ss strain.

### 2.4. Temporal Pattern of S. frugiperda Susceptibility to Teflubenzuron

The susceptibility of *S. frugiperda* to teflubenzuron was estimated by using diagnostic concentration bioassays described before. In the bioassays, approximately 480 third-instar larvae (20 replicates of 24 larvae) were tested per population. Larval survival was assessed after 5 days, using the same criteria described in Section 2.2. The temporal pattern of *S. frugiperda* susceptibility was analyzed using boxplots of larval survival data for all populations tested in each year.

For statistical analysis, we tested homogeneity with the Bartlett test and normality with the Shapiro–Wilk test of our survival data at diagnostic concentrations of *S. frugiperda* populations evaluated in each year. Since our data did not fit the assumptions for ANOVA, we performed the non-parametric Kruskal–Wallis test followed by the post hoc Dunn’s test to compare our survival data from each year using R statistical software [32].

### 2.5. Spatial Pattern of S. frugiperda Susceptibility to Teflubenzuron

To study the spatial patterns of *S. frugiperda* susceptibility to teflubenzuron, we evaluated the relationship between the latitude and longitude of each sample and the survival data from *S. frugiperda* monitoring for teflubenzuron. Data were separated into groups of two or three monitored years. Maps were created with the software QGIS 3.26.2 using a rule-based categorization of data points for the map [33]. Four categories were defined based on the survival obtained in monitoring: less than 5% survival (safe); from 5 to 20% survival (warning); from 20 to 40% (high); and more than 40% (critical). The categorization was made assuring that the points are plotted from lower to higher survival rates; this guarantees that in case of more than one point in the same place, we always show the highest survival category.

## 3. Results

**Figure 1:**
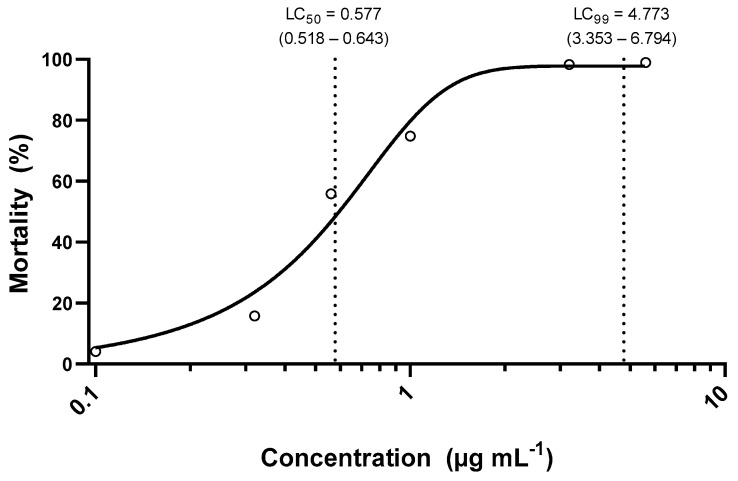
Concentration-response curve of the *Spodoptera frugiperda* susceptible strain (Sf-ss) to teflubenzuron.

The LC_50_ value estimated for Sf-ss strain was 0.577 (95% CI 0.518–0.643) and the LC_99_ estimated was 4.773 (95% CI 3.353–6.794) µg mL^−1^ (n = 789; slope = 5.01 ± 0.41; *χ^2^* = 6.81; *df* = 4) (Figure 1). The diagnostic concentration for the susceptibility monitoring of *S. frugiperda* to teflubenzuron in Brazil was defined as 10 µg mL^−1^ of teflubenzuron, based on the concentration next to the upper limit of the LC_99_ of the susceptible strain.

**Figure 2:**
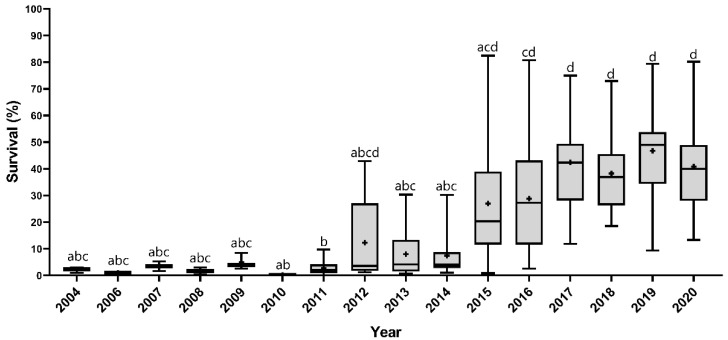
Boxplot showing the increase in survival of *Spodoptera frugiperda* populations to teflubenzuron from 2004 to 2020. The (+) sign shows the mean survival at diagnostic concentration of teflubenzuron in populations tested in that year. Different letters show a significative statistical difference between groups (Dunn’s test, *p* < 0.05).

The Kruskal–Wallis non-parametric test showed that there are significant differences between the years (*χ^2^* = 145.39; *df* = 15; and *p*-value < 0.0001). The susceptibility of *S. frugiperda* populations to teflubenzuron showed a decreasing trend over the years (Figure 2). Initially, the *S. frugiperda* survival average was less than 5% at the diagnostic concentration in all tested populations in 2004, but reached values of up to 80% in some populations over the years (Figure 2). However, this trend was not the same in all locations. After 2012, a high variation in the susceptibility to teflubenzuron was detected between populations of *S. frugiperda* from different locations (Figure 2; Table S1).

Five populations of *S. frugiperda* were evaluated in 2012. Survival rates were lower than 5% in four populations, but the population from the GO state presented a survival of ≈ 43%. In 2013, 19 populations were evaluated, and the survival rates from GO, BA, and MT states were higher than the survival rates of populations from other states. Thus, the susceptibility to teflubenzuron was reduced earlier in the states of GO, BA, and MT and later in the other states. By 2015, most of the populations presented high survival rates, regardless of the state (Table S1). Therefore, the decrease in the susceptibility of *S. frugiperda* to teflubenzuron had different spatial patterns over time. 

**Figure 3:**
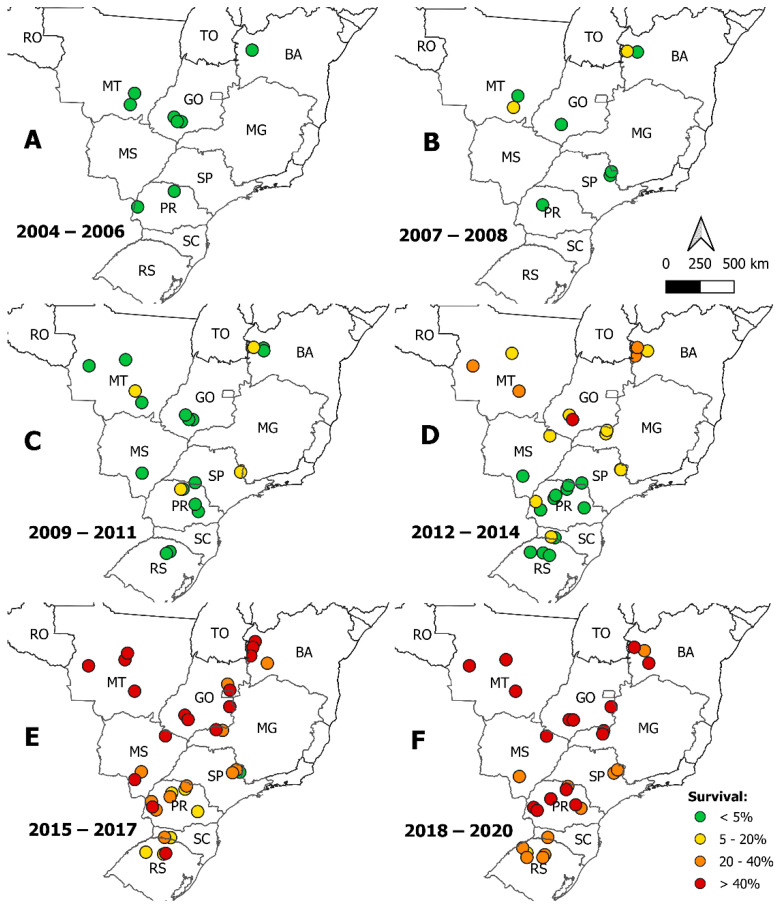
Spatial distribution of *Spodoptera frugiperda* survival to teflubenzuron at diagnostic concentrations. Each colored dot is a different tested population. (**A**) shows safe survival levels (less than 5%) in all evaluated locations; (**B**) shows an increase in survival to a warning level (5 to 20% survival) in northeast and northwest areas; (**C**) shows the warning spreading to other areas; (**D**) warning level spread to other locations (20 to 40%), and the critical level (>40%) is shown; (**E**) shows the spread of the critical level all over the country; (**F**) shows how critical survival rates are maintained over the years.

The spatial pattern of *S. frugiperda* susceptibility to teflubenzuron over time was observed by plotting the populations colored by the survival rate (Figure 3). From 2004 to 2006, it was observed a high susceptibility in all populations monitored, regardless of the state the population that was collected (Figure 3A). In 2007 and 2008, the MT and BA states started to show warning signals (Figure 3B). The population from MT state showed survival rate of ≈6%, while the population from BA states showed survival rate higher than 7% (Table S1). From 2009 to 2011, warning signals continued in the MT and BA states but also showed in populations collected in the SP and PR states (Figure 3C). The highest survival rate in this period was 8.4% for the MT population, 12.4% for the BA population, 6.9% for the SP population, and 9.7% for the PR population (Table S1).

After 2012, high and critical survival rates appeared. From 2012 to 2014, high warnings and critical signals were observed in populations from the MT, BA, GO, and MG states. The critical signal was observed in the population GO-19, with ≈43% of survival. The high signals were observed in two populations from BA states with ≈24% survival rate in each population and two populations from MT states with a 30% survival rate in each population (Table S1). During this period, populations from the SP, PR, MS, and SC states showed both safe and warning signals, while the populations collected in RS state remain in the safe categorization with <5% survival rate (Figure 3D).

From 2015 critical signals were observed in practically all populations of *S. frugiperda* evaluated from different states. All populations of MT state evaluated from 2015 to 2017 showed critical survival rates, varying from 51.6% in MT-33 to 82.46% in MT-32. Populations evaluated in the BA, GO, MS, and MG states showed high and critical survival rates in this period. The loss of susceptibility happened fast in these states; for example, in the BA state the increase in survival changed from one year to the next, as the population BA-40 showed 32.3% survival in 2017 and the population BA-41 showed 49.7% survival in 2018 (Table S1). Populations from the other states showed survival rates in all four categorizations, with one population from the SP state still presenting a low survival of 2.5% (Figure 3E and Table S1).

Lastly, from the 2018 to 2020 susceptibility monitoring years, most of the survival rates of *S. frugiperda* to teflubenzuron were higher than 20%, regardless of the state of the population collected. Populations from the MT, GO, MG, and PR states varied from high to critical survival rates, while populations from the SP and RS states showed only high survival rates (Figure 3F). The highest survival rate was observed in the MT-55 population in 2020 (80.2%) (Table S1).

## 4. Discussion

This study shows an extensive period of susceptibility monitoring of *S. frugiperda* to teflubenzuron and indicates the consequences of its intensive use for insecticide resistance management (IRM) programs. In this paper, we clearly showed the reduction in the susceptibility to teflubenzuron of the *S. frugiperda* populations over the years.

In the first years of the susceptibility monitoring program (2004–2006), the survival rates at diagnostic concentrations of teflubenzuron was less than 5% in all tested populations, and no field failures in the control of *S. frugiperda* were reported with the use of this insecticide. At that period, lepidopteran pest management was strictly performed with insecticides, because Bt corn was only approved for commercial release in 2007 in Brazil. Between the years of 2007 and 2008, warning signals were already observed, as some higher survival rates (5 to 20%) at diagnostic concentrations were registered. These first warnings appeared in the MT and BA states, the regions with the intensive use of insecticides, large corn production, and a mean annual temperature of 26–28 °C [34,35], which is in the optimal temperature range for *S. frugiperda* development [36].

The intensive agricultural systems in Brazil, with their extensive areas and overlap of crop seasons, are due to favorable soil and climate conditions in most regions. In some regions, such as parts of the BA and MT states, the succession plantation of corn, cotton, and soybean is common, and all these crops are suitable hosts for *S. frugiperda*. Furthermore, during the fallow period, it is very common to grow millet (*Pennisetum americanum* (L.) K. Schum.) as a cover crop, which is also a very good host plant for *S. frugiperda*. Thus, this pest has a constant food source. Those crops are usually sprayed with the same set of insecticides; therefore, this pest is under constant exposure to selection pressure, favoring the reduction in the susceptibility to teflubenzuron and other insecticides in *S. frugiperda* populations in Brazil over the years.

**Figure 4:**
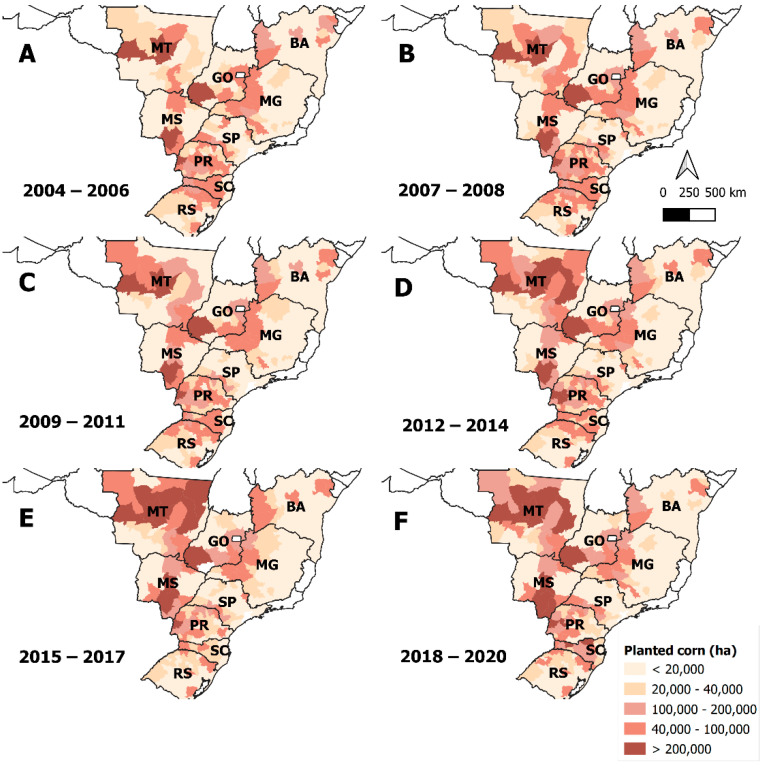
Maps showing cultivated corn areas across main corn-growing areas in Brazil. Cultivated area in each map is the mean value for the years considered in each map. Database used to build the maps can be found at this URL: https://www.ibge.gov.br/estatisticas/economicas/agricultura-e-pecuaria/9117-producao-agricola-municipal-culturas-temporarias-e-permanentes.html?=&t=destaques, accessed on 10 November 2022.

The effect of extensive agriculture on the reduction of susceptibility to teflubenzuron in *S. frugiperda* populations over time can be analyzed by considering the planted area of corn crops in a specific region. The first two signals of warning were both collected in microregions of the MT and BA states, which present large areas of cultivated corn fields with approximately 85,000 and 147,000 ha of corn per year, respectively [35] (Figure 4). On the other hand, microregions with smaller areas of corn fields presented a late reduction in susceptibility to teflubenzuron. In the following years of susceptibility monitoring, the first signals of high (20–40%) and critical (>40%) survivals were also observed in regions with large cornfield areas. The first critical survival level appeared in 2012 in a microregion with almost 750,000 ha of cultivated corn per year in the GO state, one of the regions with the largest planted corn areas in Brazil [35].

**Figure 5:**
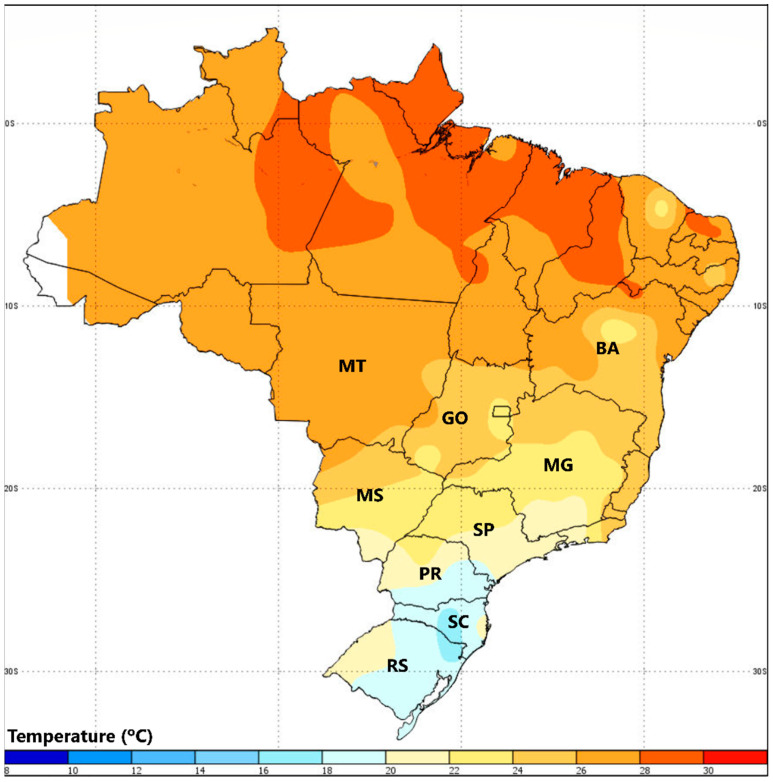
Mean annual temperature in Brazil from 1991 to 2020. Source: URL: https://clima.inmet.gov.br/NormaisClimatologicas/1961-1990/precipitacao_acumulada_mensal_anual, accessed on 10 November 2022.

Higher survival rates at diagnostic concentrations were found in microregions with higher mean temperatures (Figure 5). Temperature is known to be linked with insect population growth, which has a positive correlation with metabolic rates and a negative correlation with crop losses by insect pests [37]. The high temperatures in regions from BA and MT states, where it was first observed the survival warning signals, are optimal for *S. frugiperda* development. Thus, this pest presents more generations per year, sometimes with more than two generations in a single crop season. The high number of *S. frugiperda* generations associated with the high selection pressure associated with the use of insecticides favors selecting individuals that are more tolerant to insecticides. Additionally, higher temperatures can be associated with higher metabolic rates that can lead to increased detoxification for the pest [37]. The detoxification mechanisms have been recently associated with *S. frugiperda* resistance to teflubenzuron [28].

From 2009, warnings spread to other parts of the country, and even with the high adoption of Bt corn, the survival rate for teflubenzuron kept increasing at a fast pace, achieving critical values of up to 80% in some locations, which were maintained until the end of the susceptibility monitoring program in 2020. The decreasing trend in the susceptibility of *S. frugiperda* to teflubenzuron detected in most populations evaluated in 2015 may be associated with a higher use of benzoylurea insecticides after the detection of *Helicoverpa armigera* (Hübner) in Brazil in 2013 [38]. Several insecticides, including benzoylureas, were released as an emergency permit to control this invasive pest in Brazil at that time [39]. Both *S. frugiperda* and *H. armigera* share the same hosts niche and chemical control tactics, hence insecticide sprays to control *H. armigera* also enforced selection pressure in *S. frugiperda*. This premise that insecticides used to control *H. armigera* interfered with the susceptibility of *S. frugiperda* to insecticides was also observed in the monitoring of emamectin benzoate in field-collected populations of *S. frugiperda* in Brazil [40]. Due to recent resistance evolution to benzoylureas in some lepidopteran pests in Brazil [11,27,28,29], a reduction in the use of benzoylureas was detected in Brazil; benzoylurea insecticides were responsible for 27% of the market share of the insecticides to control lepidopteran pests in 2015 and only 18% of the market share in 2020 [19].

The reduction in susceptibility to teflubenzuron in *S. frugiperda* populations over time provides clear evidence of field-evolved resistance in Brazil. Resistance to teflubenzuron has been recently characterized as polygenic and autosomal, incompletely recessive, and suggested to be linked with detoxification mechanisms in *S. frugiperda* in Brazil [28], which corroborates with our first reports of susceptibility reduction in monitoring in warmer regions of Brazil. However, resistance due to metabolic detoxification can carry a high fitness cost associated with it [41,42]. The presence of fitness costs associated with resistance can be used in insecticide resistance management (IRM), as in the absence of insecticide selection pressure, the individuals that carry those resistance alleles have lower fitness than susceptible ones [43]. One of the strategies to manage insecticide resistance is the rotation of insecticides that do not show cross or multiple resistance between each other within or between different modes of action (MoA) [28,44]. The use of IPM measures is essential as well, we can point out the use of Bt crops, selective insecticides to keep natural enemies and to be used together with biological control with macro and microorganisms, mating disruption with pheromones and transgenic *S. frugiperda* individuals with self-limiting gene [8,45,46,47,48,49].

## 5. Conclusions

In conclusion, a reduction in the susceptibility to teflubenzuron in *S. frugiperda* populations throughout the years was documented in Brazil. Additionally, spatial variation in the susceptibility to teflubenzuron was also detected in *S. frugiperda* populations due to variations in climatic conditions and agricultural landscapes in different locations. This research provides evidence of field-evolved resistance of *S. frugiperda* to teflubenzuron and reinforces that IRM strategies are urgently needed to be implemented in Brazil.

## Data Availability

The data presented in this study is available in Table S1.

## Supplementary Materials

The following supporting information can be downloaded at: https://www.mdpi.com/article/10.3390/insects14020129/s1. Table S1: survival rate (%) of *Spodoptera frugiperda* at diagnostic concentration of teflubenzuron in field-collected populations between 2004 and 2020.
